# Late-Onset Manic Episode with Psychotic Features Associated with a Posterior Fossa Arachnoid Cyst

**DOI:** 10.1192/j.eurpsy.2026.11049

**Published:** 2026-07-31

**Authors:** M. Mouldi, M. Khamassi, H. Ghabi, M. Karoui, H. Nefzi, R. Kamoun, F. Ellouze

**Affiliations:** Department of Psychiatry G, Razi Hospital, Manouba, Tunisia

## Abstract

**Introduction:**

Arachnoid cysts are benign, cerebrospinal fluid-filled lesions representing around 1% of intracranial space-occupying processes. They are mostly asymptomatic, yet some studies have described psychiatric manifestations such as mood instability, cognitive deficits, or psychosis. Posterior fossa arachnoid cysts (PFACs) are particularly rare and more often linked to cerebellar or vestibular symptoms. Recent findings emphasize the cerebellum’s role in emotional regulation and its contribution to affective and psychotic disorders.

**Objectives:**

This study aims to report a case of late-onset mania with psychotic features associated with a posterior fossa arachnoid cyst and to explore the potential neuropsychiatric implications of such lesions.

**Methods:**

We report the case of Mr. X, a 53-year-old retired army corporal with no personal or family psychiatric history, who was admitted for acute behavioral disturbances. The clinical evaluation focused on identifying manic and psychotic symptoms, and a full neurological workup was performed.

**Results:**

The patient presented with a euphoric and irritable mood, psychomotor agitation, pressured speech, and a decreased need for sleep. He exhibited grandiose delusions as well as persecutory and jealous delusions. The clinical picture was also marked by auditory and visual hallucinations. This constellation of symptoms met the diagnostic criteria for a manic episode with psychotic features. The Young Mania Rating Scale (YMRS) score was 21, confirming a state of significant manic excitement. The neurological examination was notable for a slight unsteadiness on tandem gait. Cerebral CT and MRI revealed a 21 × 30 mm retrocerebellar arachnoid cyst compressing the cerebellum with a mild mass effect. EEG and standard laboratory tests were within normal limits. Treatment with risperidone was initiated, leading to a gradual but significant clinical improvement over the following weeks.

**Conclusions:**

This case highlights a possible association between posterior fossa arachnoid cysts and late-onset affective-psychotic symptoms. It underscores the importance of considering organic etiologies, including structural cerebellar abnormalities, in atypical or late-onset psychiatric presentations. Further studies are needed to clarify the pathophysiological role of the cerebellum in neuropsychiatric syndromes.

**Disclosure of Interest:**

None Declared

